# Implementing body composition assessment into clinical practice in patients with acute spinal cord injury- a pilot feasibility study

**DOI:** 10.1038/s41393-026-01169-2

**Published:** 2026-02-02

**Authors:** Katherine J. Desneves, Bryn Fittall, Chantelle Elson, Robin M. Daly, Leigh C. Ward, Nicole Kiss

**Affiliations:** 1 Department of Nutrition and Dietetics, Division of Allied Health, Austin Health, Studley Rd, Heidelberg, 3084 Victoria Australia; 2 Institute for Physical Activity and Nutrition (IPAN), Deakin University, Geelong, 3125 Australia; 3 Department of Physiotherapy, Division of Allied Health, Royal Talbot Rehabilitation Centre, Austin Health, Yarra Boulevard, Kew, 3101 Australia; 4 School of Chemistry and Molecular Biosciences, The University of Queensland, St Lucia, 4072 QLD Australia; 5 Allied Health Research, Peter MacCallum Cancer Centre, Melbourne, 3000 Australia

**Keywords:** Nutrition, Spinal cord diseases

## Abstract

**Study design:**

Prospective mixed methods implementation study.

**Objectives:**

To: (1) implement a SCI-specific care pathway for body composition assessment (ATSCI-Nut); (2) pilot test the feasibility (reach, adoption, adherence, appropriateness, and acceptability) of the care pathway in patients with new traumatic SCI; (3) explore patient experiences with the care pathway and the effect of providing body composition information on dietary choices and (4) explore clinician experiences with the new care pathway.

**Setting:**

Victorian Spinal Cord Service, Australia

**Methods:**

Participants included individuals with acute SCI who received the ATSCI-Nut pathway and consented to data collection. Feasibility outcomes (reach, adoption and intervention fidelity) were collected from medical records. Acceptability and appropriateness were explored via patient semi-structured interviews and clinician focus groups.

**Results:**

Twenty-three patients were eligible, 21 (91%) consented. Adherence to the ATSCI-Nut pathway initial assessment and review components during weeks 2–8 and >8 weeks was 86, 71 and 69%, respectively. Adherence to completing bioimpedance spectroscopy (BIS) measurements at specified time-points was 69%. However, only 43% of participants had all BIS measurements completed at specified time-points. Two themes were common to patients and clinicians: physiological and body composition changes directing focus of rehabilitation, and barriers and enablers to optimal care. One additional theme arose from patient interviews: impact of SCI on self-image and lifestyle.

**Conclusions:**

The ATSCI-Nut pathway is a feasible and acceptable model to deliver body composition assessment despite mixed adherence to the pathway overall. However, barriers to optimal patient care and pathway adaptations need to be explored to improve adherence.

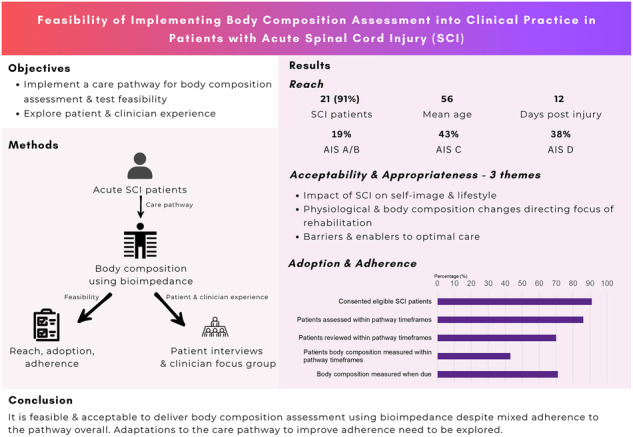

## Introduction

Increased adiposity and decreases in muscle mass and energy requirements occur following acute spinal cord injury (SCI), defined as the acute and rehabilitation hospital admissions immediately after injury [1, 2]. Body composition changes differ according to neurological level of injury and extent of sensory and motor impairment [1]. During the acute phase, a diet adequate in energy and protein is advised to avoid undernutrition, while during rehabilitation, a healthy lifestyle is encouraged to avoid weight gain [3]. However, body weight is a crude measure that doesn’t distinguish between muscle and fat. A 2022 review and clinical practice guidelines published in 2018 recommend assessing resting metabolism using indirect calorimetry (IC) [1, 4] or using SCI-specific energy prediction equations when IC is unavailable [1], and monitoring body composition [1, 4] at least annually [1]. Previous guidelines from 2009 recommend assessing body composition using bioimpedance or dual energy x-ray absorptiometry (DXA) [3]. However, the guidelines do not stipulate when and how often to repeat body composition measurements, nor how to interpret and use the results in clinical decision making during the acute and rehabilitation phases after SCI.

Care pathways describe activities, content and frequency of care that patients should receive throughout the care continuum [5]. Benefits of care pathways include translation of evidence into practice, standardised care, reduced practice variation and improved patient care, safety, and outcomes [5]. The Action, Actor, Context, Target and Time (AACTT) framework can assist in care pathway design by specifying who should do what, where, for whom and when and to help characterise actions that could be measured to assess uptake and/or adherence [6].

Bioimpedance is a valid method for assessing body composition [fat-free mass (FFM) and fat mass (FM)] during the acute and rehabilitation phases after SCI [7] and this FFM measure can be used in SCI-specific energy prediction equations to inform individual energy needs [8–10]. After SCI, we have shown that body fat changes over time in a U-shaped trajectory, initially decreasing over the first 3–4 months and returning to baseline by 7 months [11]. These key time points were used to develop an Acute Traumatic Spinal Cord Injury Nutrition (ATSCI-Nut) Care Pathway to standardize care and translate the use of bioimpedance and a SCI-specific energy predication equation [8] into clinical practice and to guide clinical decision making about when to change the dietary intervention focus from prevention of undernutrition to health promotion. This study aimed to: (1) implement a SCI-specific care pathway for patients with new traumatic-SCI; (2) test the adoption, feasibility, adherence, appropriateness and acceptability of implementing bioelectrical impedance spectroscopy (BIS) to assess body composition and a SCI-specific energy prediction equation into clinical practice; (3) explore patient experiences with the new care pathway and patient perceptions of the effect of providing information regarding body composition on dietary choices, and to (4) explore clinician experiences with the new care pathway.

## Methods

### Study design and sampling pool

This was a single-site pilot prospective implementation study. A mixed methods approach guided by the theoretical framework of acceptability [12] and the implementation framework proposed by Peters et al., [13] was used to evaluate pathway implementation outcomes. The sampling pool included staff who delivered care and patients who received care according to the pathway. Evaluation included collection of implementation and outcome information, patient demographic and clinical data and patient semi-structured interviews, a staff survey and staff focus group.

### Setting

This study was conducted across the acute and rehabilitation wards of the Victorian Spinal Cord Service, a statewide service for individuals who experience traumatic SCI. Care pathway components were delivered based on scheduled timeframes rather than ward location, as patients moved variably between acute and rehabilitation settings- including possible transfers back to acute care.

### Ethics approval and consent

Ethics approval was received from the Human Research Ethics Committee (HREC/75733/Austin 2022). Patients gave verbal, witnessed informed consent to participate in the care pathway and additional verbal consent to participate and for audio recording of the interview. Clinician participants in the focus group provided written informed consent and completion and return of the survey implied clinicians’ consent. All methods were performed in accordance with the relevant guidelines and regulations.

### Development of the ATSCI-nut pathway

The project team [dietitians, exercise physiologist (EP) and body composition experts] designed the ATSCI-nut care pathway based on clinical practice recommendations, guidelines [1, 3, 4] and recent research [7, 9–11]. The AACTT framework was used to help define behaviours and components of care within the pathway [6].

Components considered within the pathway to support initiation of appropriate clinical care processes and sustainable implementation included:

- screening and referral processes;
- setting specific actions or behaviours;
- specifying who is to conduct each action and on whom the actions are performed;
- timelines for clinical processes including screening, assessment and treatment and; definition of assessments to be administered when and by whom.

**Fig. 1:**
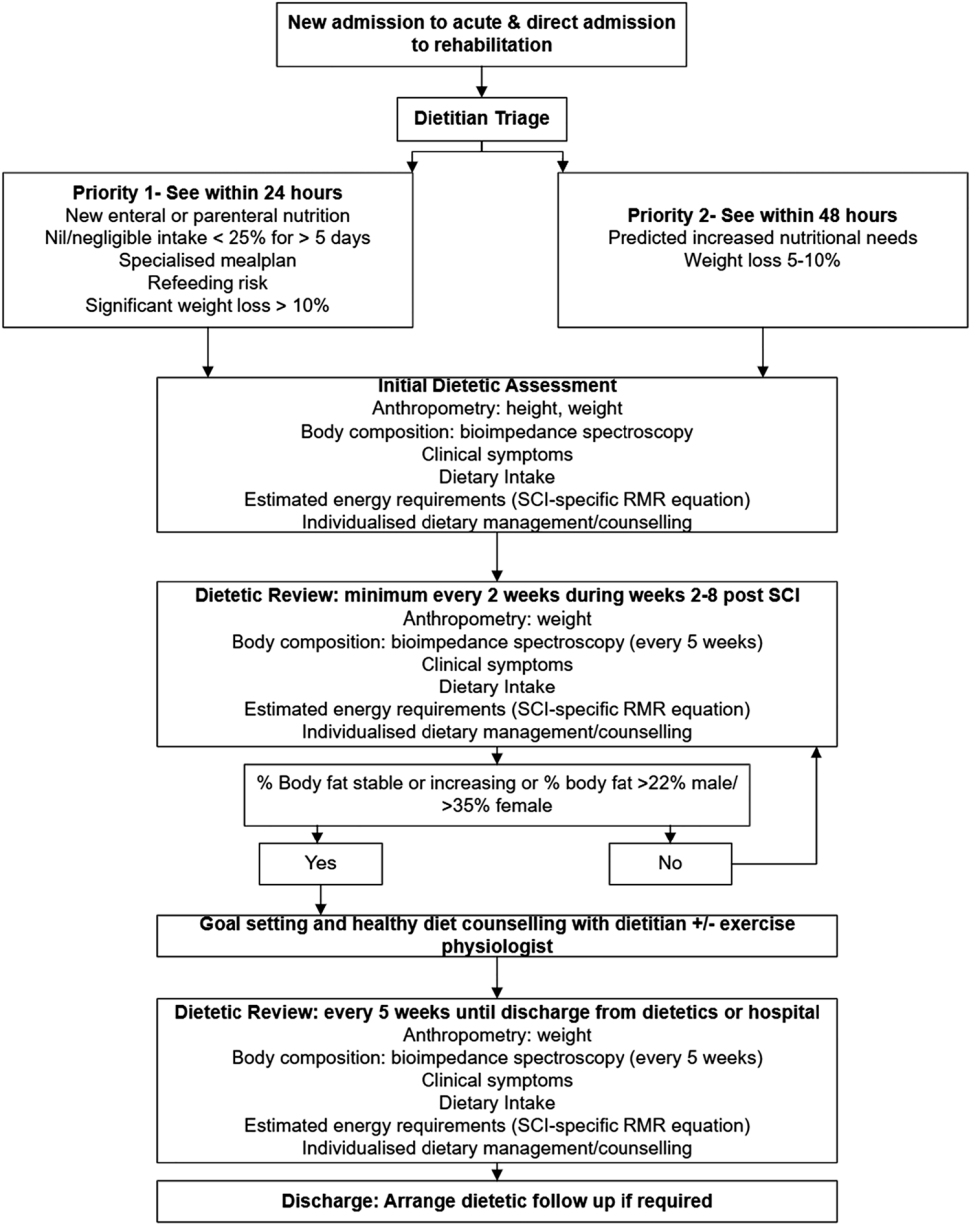
Acute Traumatic Spinal Cord Injury (ATSCI-Nut) Pathway.

Local barriers and enablers to implementing the pathway were identified and considered. Supplementary Table 1 summarises clinical practice guidelines, and the recent literature mapped to the AACTT framework [6]. Figure 1 illustrates the ATSCI-nut care pathway.

### Implementation strategies

The implementation strategies utilised are reported according to the Expert Recommendations for Implementing Change (ERIC) study classifications [14].

#### Train and educate stakeholders

Dietitians and an EP completed an education program and a BIS competency package developed by the study lead. This included an overview of the care pathway and BIS methodology, observing the study lead conducting BIS, completing BIS with the study lead, followed by independently completing BIS with the study lead observing.

#### Support clinicians

The study lead was present for BIS measurements until the staff member felt confident and was competent to complete measurements independently.

#### Develop stakeholder interrelationships

The project team met monthly during the first six months of implementation. The multidisciplinary ward newsletter and Quality Improvement Committee were used to communicate project updates prior to study commencement, at the end of recruitment and study cessation.

#### Use evaluative and iterative strategies

A continuous quality improvement feedback cycle was used to make immediate adjustments to the care pathway processes.

#### Change infrastructure

The care pathway was included in a local nutrition clinical guideline. Additional strategies used after the initial implementation included development of a spreadsheet to track dates body composition measurements were due and clarification of processes when patients transferred between campuses.

#### Engage consumers/adapt and tailor the content

Plotting and printing body composition trends was introduced following patient reports of poor communication about body composition results.

#### Pilot test the feasibility of the ATSCI-nut care pathway

The care pathway was piloted between May 2023 and October 2024 without additional staffing resources. The duration of the pilot was determined by number of eligible patients and available staffing resources. Operational and clinical data were entered into the Research Electronic Data Capture (REDCap) secure web platform [15] by a project team member weekly.

#### Participants

All patients >18 years with a new traumatic-SCI admitted between May 2023-January 2024 were eligible. Patients were excluded if they had a pacemaker, were pregnant or breastfeeding, in ICU or had a concurrent severe acquired brain injury (post-traumatic amnesia duration >7 days). Eligible patients were approached and gave verbal consent to receive clinical care, as per the care pathway. Twenty-one participants undertook individualised rehabilitation as per Clinical Practice Guidelines [16] and 30–60-min of exercise training (supported by an EP) 2–3 days per week including weights and/or wheelchair skills except for 3 patients who were also participants in an intensive rehabilitation trial. All patients received the diet prescribed by their treating dietitian. BIS measurements were scheduled on patient rehabilitation timetables. Individualised body composition feedback to patients was integrated into face-to-face dietitian reviews or goal setting and healthy lifestyle counselling delivered by the dietitian and/or EP.

Six clinicians (5 dietitians and 1 exercise physiologist) who delivered care according to the care pathway between May 2023 and October 2024 were eligible and participated in the study.

#### Patient demographic and clinical and body composition data

Participant demographics, clinical characteristics and admission American Spinal Injury Association (ASIA) Impairment Scale (AIS) criteria [17] were collected prospectively from the electronic medical record (EMR). Weight and supine length were obtained as described previously [7].

Body composition measurements were taken, either before scheduled exercise physiology sessions or in the morning whilst participants were in bed, using a four terminal BIS instrument (SFB7, ImpediMed. Brisbane, Australia) whilst the patient was supine as previously described [7]. The BIS instrument was set to selected frequency mode and a single whole-body measurement was taken on the right side of the body at 50 kHz. Participants were advised to eat and drink as usual, empty bladder if voiding on sensation and avoid caffeine and exercise two hours before the measurement. Body composition (FFM and FM, percentage body fat) were calculated in REDCap using an SCI-specific equation FFM equation [18]. Individual energy requirements were also calculated in REDCap using a SCI-specific energy prediction equation [8] and injury and/or activity factors [19–21] deemed clinically appropriate by the dietitian. Data on nutritional goal setting and healthy diet counselling were gathered retrospectively from the EMR.

#### Staff demographic data

Demographic data on age, sex, occupation, duration of working with patients with SCI and previous experience using bioimpedance were collected via a staff survey in REDCap [15].

#### Adoption

The study lead collected data on the number of patients (i) admitted during the pilot study (ii) who were eligible, approached and consented to receive care and (iii) received care according to the care pathway.

#### Intervention delivery and adherence

Data collected included number and proportion of:

- priority one patients (new enteral or parenteral nutrition, nil/negligible intake <25% for >5 days, specialised mealplan, high refeeding risk, significant loss of weight ≥ 10%) seen within 24 h;
- priority two patients (predicted increased nutritional needs, ≤ 50% intake for >5 days, loss of weight 5–9.9%) seen within 48 h;
- patients assessed and reviewed at all timepoints as per pathway timeframes;
- patients who had assessments, reviews and clinical assessment measures performed as per the care pathway, including FFM and FM using BIS and energy requirements calculated using the SCI-specific Buchholz et al., [8] energy predication equation;
- patients where percentage body fat guided a change in dietary management.

Adherence was classified as high (80–100%), moderate (50–79.9%) and low (<50%) [22].

#### Participant acceptability and appropriateness

Patient acceptability and appropriateness of the care pathway was assessed using semi-structured interviews in a subset of participants before discharge from rehabilitation. Patients were invited to participate in an interview with the study lead, gave verbal consent to participate and for audio recording of the interview. Sampling was purposive and aimed to recruit 10–15 patients of diverse age, sex and injury severity to gain a broad understanding of acceptability and appropriateness. The interview guide was composed of predetermined semi-structured questions and accompanied by follow-up probes and questions that emerged from the interview (Supplementary Table 2). Interviews were conducted in person with only the interviewer (study lead - trained in qualitative research methods) and participant present (one patient’s spouse was present) and lasted 10–33 min.

A focus group was conducted with clinicians who provided clinical care following the care pathway using a semi-structured interview guide in May 2024 (Supplementary Table 3). Clinicians provided written and verbal recorded consent for interview participation. The staff focus group was conducted, recorded and transcribed via Microsoft Teams and lasted 51 min. The interviewer had a pre-existing clinical relationship with most patients and a pre-existing professional relationship as a senior colleague with all clinicians.

The interview and focus group guide questions were developed based on the study aims, existing literature [1, 3, 4, 9–11, 23] and the key dimensions of acceptability [13, 24]. Each interview and the focus group were electronically audio recorded, de-identified (using numerical coding) by the study lead who conducted the interview; and transcribed verbatim using artificial intelligence software (Otter.au Los Altos, CA). The transcriptions were verified for accuracy against the audio recording by the study lead and corrected as required. All participants were offered the opportunity to check transcripts for accuracy.

### Data management and analysis

#### Demographic, clinical and operational data

After data entry into REDCap was completed, data were exported into Excel and/or Stata/BE 17.0 (College Station, TX) for analysis. Descriptive statistics including medians and interquartile ranges were used for continuous variables due to non-normal data distribution. Categorical variables and intervention delivery and adherence were reported as counts and proportions.

#### Patient interviews and staff focus groups

Interview and focus group transcripts were managed using NVivo 14.23.3 to facilitate thematic analysis [25]. Data collection and analysis for the patient interviews were completed simultaneously. Recruitment ceased when data saturation was reached. Five clinicians participated in the focus group. One researcher (KD) read and corrected interview and focus group transcripts in their entirety and completed initial open coding, which were reviewed by a second researcher (NK) after four interviews and at the completion of all interviews. Key concepts were identified from the data using an iterative and reflective process [25]. The two researchers then grouped the codes into themes.

## Results

### Staff characteristics

Six clinicians (1 male) comprising five dietitians (including study lead) and one EP completed BIS assessments. Clinician median age was 46 years (IQR 36, 58). Years of experience working with patients with SCI varied; < 1 year (n = 2),1–2 years (n = 1), 5–10 years (n = 2), and > 20 years (n = 1). Two staff had experience using bioimpedance.

### Adoption

Thirty-four patients with a new acute traumatic-SCI were admitted and 23 were eligible for inclusion in the study. Eleven patients were ineligible: severe acquired brain injury (n = 6) and ventilator-dependent (n = 5). Ninety-one percent of the 23 new acute traumatic-SCI persons who were eligible consented (n = 21). One patient declined participation, and one patient was unable to be approached for consent.

### Reach

Participants median age was 56 (IQR 54, 64) years, median time post-injury 12 days (IQR 6, 16), 76% were male and 81% had tetraplegia. Four participants with tetraplegia and one participant with paraplegia had motor complete SCI (AIS A-B) (Table 1). Median BMI at initial assessment was 24.8 (IQR 23.5, 29.1) kg/m^2^. 48% of participants were overweight or obese using WHO BMI cut-offs of >25 kg/m^2^ and >30 kg/m^2^ respectively [26].

**Table 1:** Baseline demographic and body composition characteristics of 21 participants with spinal cord injury (SCI).

Characteristic	All participants	Males	Females
Participants, n (%)	21	16 (76)	5 (24)
Age (years)	56 (45, 64)	58 (46, 65)	47 (43, 58)
Injury, n (%)			
High tetraplegia (C1-C4), n (%)	14 (67)	11 (69)	3 (60)
AIS A/B	3 (14)	2 (13)	1 (20)
AIS C	5 (24)	5 (31)	
AIS D	6 (29)	4 (25)	2 (40)
Low tetraplegia (C5-C8), n (%)	3 (14)	2 (13)	1 (20)
AIS A/B	1 (5)	1 (6)	
AIS C	1 (5)		1 (20)
AIS D	1 (5)	1 (6)	
High paraplegia (T1-T7), n (%)	2 (10)	2 (13)	
AIS C	2 (10)	2 (13)	
Low paraplegia (T8-L2), n (%)	2 (10)	1 (6)	1 (20)
AIS C	1 (5)		1 (20)
AIS D	1 (5)	1 (6)	
Days post injury	12 (6, 16)	13 (7, 30)	11 (6, 13)
Weight (kg)	75.9 (66.6, 83.0)	78.2 (69.4, 85.1)	66.5 (64.4, 77.3)
Height (cm)	176.0 (164.5, 185.0)	179.0 (171.0, 186.0)	161.0 (161.0, 164.0)
BMI (kg/m^2^)	24.8 (23.5, 29.1)	24.8 (23.0, 28.6)	24.8 (24.7, 31.0)
Normal BMI 18.5–24.9, n (%)	11 (52.3)	8 (50)	3 (60)
Obese BMI ≥ 25, n (%)	10 (47.6)	8 (50)	2 (40)
Body Composition (Bioimpedance)			
Total body FFM (kg)	54.3 (46.9, 57)	55.8 (49.9, 62.3)	43.9 (43.4, 46.7)
Total body FM (kg)	21.6 (19.7, 26.1)	21.05 (19.2, 25.6)	23.6 (19.8, 33.4)
% Total body fat	29.1 (26.5, 36.4)	28.0 (25.3, 33)	36.6 (29.8, 43.2)
Estimated Energy Requirements (MJ)	7.4 (6.7, 8.5)	7.8 (7.2, 8.9)	6.6 (6.5, 7.2)

Values are numbers and percentages or median and interquartile ranges.

*AIS* American Spinal Injury Association (ASIA) Impairment Scale (AIS), *BMI* body mass index, *FFM* fat free mass, *FM* fat mass, *cm* centimeter, *kg* kilogram, *MJ* megajoules.

### Intervention delivery and adherence

Adherence to initial assessment timeframes outlined in the care pathway was high. Eighty-seven percent of priority 1 and 83% of priority 2 patients were assessed within 24 and 48 h of admission (Table 2). Participants had a mean of 3.00 ± 1.23 BIS measurements (range 1–5) completed. Four participants had one BIS measurement (2 discharged home, 1 transferred to general rehabilitation, 1 not participating in rehabilitation). The median time on the care pathway was 154 days (IQR 113, 179). Adherence for completing dietetic reviews as per pathway timeframes was moderate (71% reviewed fortnightly during weeks 2–8, 69% reviewed every 5 weeks until discharge from dietetics, discharge from or cessation of active rehabilitation). Adherence to completing BIS measurements when due was moderate (69%). Only 43% of participants had *all* BIS measurements completed within pathway timeframes, indicating low adherence to the pathway overall. The main reasons for non-completion of BIS measurements and estimation of energy requirements were patient unavailable (28%), BIS measurement inaccurate in one undernourished patient (18%), bed rest due to pressure injury (14%), clinician unplanned leave (11%) or patient unwell (11%). Goal setting and healthy diet counselling were initiated for 71% of participants. Percentage body fat guided a change in dietary management for 57% of participants.

**Table 2:** Adherence to time and clinical assessment measures within the ATSCI-Nut Pathway.

Components of the ATSCI-Nut Pathway	Number of patients (%)
**Adherence to pathway timeframes**	
Assessment within pathway timeframes (n = 21**)**	18 (85.7)
Priority 1 patients seen within 24 h (n = 15)	
Completed	13 (86.7)
Not completed	2 (13.3)
Priority 2 patients seen within 48 h (n = 6)	
Completed	5 (83.3)
Not completed	1 (16.7)
Review within pathway timeframes	
Fortnightly (weeks 2–8) (n = 21)	
Completed	15 (71.4)
Not completed	6 (28.6)
Every five weeks ( > 8 weeks - discharge) (n = 16)	
Completed	11 (68.75)
Not completed	5 (31.25)
Discharged < 5 weeks	3
Clinical assessment measurements within pathway timeframes (n = 21)	
Body composition & estimated energy requirements	
Completed	9 (42.9)
Not completed	12 (57.1)
Body fat % guided dietary management change (n = 21)	
Yes	12 (57.1)
No	9 (42.9)
Goal setting and healthy diet counselling (n = 21)	
Completed	15 (71.4)
Not completed	3 (14.3)
Inappropriate	3 (14.3)
Dietetic follow up arranged (n = 21)	
Yes	4 (19)
No	15 (71)
Not applicable- still inpatients	2 (10)
Median days on care pathway (IQR)	154 (113, 179)
**Adherence to BIS measurements**	**Number of measurements (%)**
BIS measurements performed when due (n = 90)	
Completed	62 (68.9)
Not completed	28 (31.1)
Patient medically unwell	3
Bed rest secondary to pressure injury	4
Patient on leave	2
Patient unavailable	8
Staff unplanned leave	3
Staff planned leave	1
BIS measurement inaccurate- malnutrition	5
Other	2

*BIS* bioimpedance spectroscopy, *ATSCI-Nut* acute traumatic spinal cord injury nutrition pathway, *IQR* interquartile range.

### Participant and clinician acceptability and appropriateness

Twelve patients (4 female) were interviewed (Supplementary Table 4), and five clinicians (4 dietitians and 1 EP) participated in the focus group. Three key themes were identified from the interviews and focus group. One theme was unique to patients: (i) impact of SCI on self-image and lifestyle whist the other two themes were common to patients and clinicians; (ii) physiological and body composition changes directing focus of rehabilitation and (iii) barriers and enablers to optimal care. Table 3 summarises patient and clinician themes, codes and quotes. Interviews and the focus group suggested the care pathway was appropriate and acceptable albeit opportunities for improvement were identified.

**Table 3:** Themes, Codes and Exemplar Quotes from the 12 Patient Interviews and Staff Focus Group.

	Patients (n = 12)		Staff (n = 5)	
Theme	Code (n = 23)	Exemplar Quotes	Code (n = 11)	Exemplar Quotes
Impact of SCI on self-image and lifestyle	Weight changes and muscle loss	“…because I was not moving…basically for the first two months, I definitely put on weight……….but I’ve just lost all the muscle” [P6] “…I lost 11 kg in the first …three weeks ….I’m guessing a lot of muscle…” [P3]		
	Impact of muscle loss on physical performance	“[muscle] not as strong. I haven’t got the strength I had before and my durability and my ability to power isn’t there” [P8]		
	Perceived protective effect of prior health and fitness	“…because I was reasonably fit before. So far I’ve maintained [body composition]… relatively good” [P4]		
	Concerns about unwanted weight	“For me I’m very conscious of not putting on weight. So that’s probably one of the biggest things for me” [P1] “…I guess I’m glad that I wouldn’t have wanted to put on weight because….I would have to …lift and move. So it’s been really critical to me and my mental health….. Just having the injury itself…it’s obviously a massive mental blow and then,,.on top of that I would not want to…….become obese…” [P3]		
	Body image concerns	“I haven’t put …weight around the stomach or anything like that” [P4]		
	Clinician discussions reinforcing patient observations	“I suspected all those things but the [body composition] measurement actually made it quite clear…..” [P7]		
	Impact of rehabilitation on body composition	“…expect that the loss of fat is going to be beneficial in the long term, but the loss of body weight I have to correct that, I have to at least stop the loss of muscle….stop it getting any worse because it’s going to have a big effect long term. Currently I’m in rehab and trying to address that issue” [P7]		
Physiological and body composition changes directing focus of rehabilitation	Impact of providing body composition information	“I now know quite clearly, what my body compositions doing and the dietitians pointed out what I need to do to augment or reverse it” [P7] “pretty early it was pointed out…that I was gonna lose protein and then the muscle tone … I think the nutritionists here did a good job informing me. I knew at the very start that I had to lose weight and I needed to be very careful about how I did it so I didn’t lose muscle mass” [P7]	Body composition and energy requirement information supporting patient care	“…it helped having this data to support some of the usual education that we provide…to have a really clear picture of what’s going on…” [HP1] I used it to inform counselling for patients that were…putting on body fat and to support the education sessions that we were already doing… it really offered that extra piece of information to support the counselling” [HP2] “…it was useful to give it in the context of the education sessions … because then that helped to frame our advice and recommendations and the education with here’s your body composition… I think the context of presentation also made a difference for how meaningful it was to the patient and potentially on board they were with some of the education recommendations.” [HP2] “I found it really useful in terms of the relationship with the patient being able to talk through things while you’re doing the measurements and getting to know them at a different level …the follow through afterwards with giving them the results.I think really enhanced our practice … I found it really useful from both the personal perspective and also the all the patients were very appreciative of the information that was given [HP3]”
	Perceived benefit of nutrition to support rehabilitation	“[nutritional considerations] as far as making sure that I’m not having an unhealthy diet, it’s going to put fat on… and that if I’m doing exercise that I’m actually building muscle mass.” [P4]	Body composition information supporting clinician decisions	“…as patients moved along in their rehab journey… the [other] predictive equations were over predicting how much energy they might need based on the bioimpedance results… that was helpful from a clinical point of view for framing my thinking around what kind of counselling I give them or what recommendations I might make because I use the requirements for framing my own thinking around what advice…using that to adjust my clinical advice to the patient, I found quite useful.” [HP2] “It’s a great tool to have in this environment to make sure that what we are doing is correct and then we’ve got the confidence to be able to educate the patients.” [HP4]
	Body composition information as a source of motivation	“…that was … inspiring to me that was nice to know that even though I’ve put on weight it was majority muscle. So that was good to know… because I’ve been doing more gym work and walking now…. that was inspiring, encouraging” [P6]	Body composition information as a source of motivation	“…if I was seeing that somebody was gaining muscle, I used that to reinforce all the positive things that they were doing.” [HP2] “…an example of a patient who was gaining some body fat, that might be a pathway to get some buy in around just generally increasing physical activity and talking about that as part of the strategy for increasing energy expenditure. So it is nice to have some numbers … to fall back on … otherwise we’re often a bit limited to talking in more general terms that might not generate the same buy in …” [HP1]
	Adjusting dietary pattern to minimise weight gain	“I have to cut back particularly… the overall proportions of food. I need to do the Mediterranean style diet much more. I need to eat more protein. Less carbohydrate. More vegetables. And fruit possibly and I need to eat less” [P7] “I’m eating less than before. Yeah, so I’m not eating as much and I’m not eating as many carbs as well.” [P11]		
	Perception that diet is more important after SCI	“Well I need to watch my diet more carefully. I can’t eat like I used to eat.” [P7]		
	Body composition information influencing food choice	“..doing the body composition test, I realized how much fat or protein I have lost or gained… that helped me to adjust my diet…if I realized that I’ve lost a considerable amount of protein, so I tried to include more protein in my diet… I try to because based on my new body condition, I try to minimize the amount of calorie or my calorie intake because I know…I don’t have much options… in terms of the physical activity, I am very limited now.” [P5]		
	Role of diet to support bowel routine	“[dietary considerations] make sure that I receive a lot of fibre for bowel for bowel management.” [P5]		
	Awareness of the importance of protein	“[nutritional considerations] I need to eat more protein.” [P7] “…advised to eat more protein based group like milk, the yogurt the custard. The fortisip, meat you know, you need to build up your muscle.” [P10] “I guess like protein …for muscle mass.” [P11]		
Barriers and enablers to optimal care	Food as a source of pleasure	“….food is one of the few things that most of us even quads can still enjoy, like flavors and tastes….” [P3]	Clinician acceptability and appropriateness	“[BIS measurements]… like anything at the start, it probably feels like a fair bit of extra work umm, working out some of your processes and … practical things like when to go see people and how the machine works and then once you’ve got all that figured out with a little bit of trial and error, I didn’t find it particularly burdensome.” [HP1] “providing feedback to that group [high quads] was the most difficult because it was much more like this is what we would expect to happen…this is what’s happening and you can’t do anything about it except change your diet, which is one of the only things that you can control now…I found that feedback really challenging to provide to patients and I felt like it wasn’t particularly useful for them” [HP2]
	Limitations of hospital food quality and variety	“… I don’t think there is enough varieties for people that are there for months on end, it is inadequate. ….there’s a disconnect between what’s provided and ….what’s appropriate…” [P4]	Clinician barriers & challenges to pathway adherence	“when it’s only like one or two a week, I think it fits in quite well.” [HP1] “…scheduling patients with a weight on that day when they’re free and when they’re happy to see you… it’s getting the planets to align…it can be a bit challenging” [HP2] “…coming from the cover [staff] perspective where it wasn’t something that you sort of doing consistently…it was that little bit more challenging because I wasn’t doing it regularly enough to probably really feel confident.” [HP5] “…I think one of the … challenges…was actually finding time to provide the feedback because it was never urgent and I always had other clinical priorities” [HP2] “[time]…because you can’t do it on the same day… you have to get the measurement, then you have to get the weight, then you have to enter it into get the system, make the graph printed off, take it to the patient…I don’t think I ever … did the measurement and the feedback on the same day. And so I would usually schedule a review the following week to feed that back, but then that would always be the review that got bumped unless there was a clinical need to see the patient… that was a challenge for me, just actually getting to provide the feedback” [HP2]
	A need for more individualised management	“I have been asking dieticians for advice on how much … portion sizes would be correct for me in my new situation, but it’s been quite vague and unhelpful such as you eat what you’re comfortable eating, the amount wise. …I was constantly wondering how much was correct for me because I didn’t have much of an idea of how much I ate before my accident… I was a.. grazer … so I have no idea how much my calories might count… [Dietitians] should give you more constructive feedback. Whether that’s alright or that’s a bit too much. Thinking of your body size and type or whatever. Because yeah it seems to be kind of vague” [P12]	Adaptations to care pathway	“…if we’re gonna continue with this [BIS] it would be figuring out who the real clinical priorities are that are going benefit from having these measures done” [HP1] “…the group that it is mostly positive news for the incomplete injuries where they’re experiencing long neurological and functional recovery because …that’s a really positive picture…we would still talk about the risk of weight gain in the longer term, but often it’s good news on the results” [HP1] “… for the really high complete quads their journey is …more predictable …there’s not the capacity to … regain any muscle mass or change their body composition through exercise… I don’t know if that would be a high value group when we’re looking at using limited resources to do the BIS… the value is not as much with that group because they can’t physically engage in exercise that’s going to maintain their muscle mass… we can predict what’s going to happen without using the BIS.” [HP2] “…exception … about the high quads. There’s one intervention that …we think can affect people’s body composition … functional, electrical stimulation cycling.… if we … knew … early on that we have this person who’s interested and appropriate and they’re likely to complete it fairly regularly, particularly if they have good family support, we sometimes train families up to help people with that…the BIS data would be quite interesting because we could actually see if that intervention is impacting their body composition in a in a positive way, particularly for that group that doesn’t have any other means of exercising.” [HP1]
	Lack of shared decision making with health professionals	“…other patients were getting protein drinks I just ate what was there. I didn’t ask any questions. And I just felt like I was making the decisions myself, I suppose.” [P4]	Importance of teamwork	“… was instrumental in getting …weights done and scheduling…also printed the scheduling…if one person was responsible for doing all of it, it would and it would have been more burdensome…because we were sharing that responsibility and it made it much more manageable.” [HP2]
	Poor communication of body composition results	“I know was initially measured [body composition] and then I’ve had what only two measurements … an interim one and then a final one. That interim one. I didn’t really ever see any feedback from it to tell me what percentage of body mass it was…” [P6]	Timely communication of body composition results	“…being able to do it in real time with the patients… having like a mobile device or something where I could just enter the data and then pull it up and take it to the patient and scroll through and go, here’s what you’ve what you’ve, uh, what we’ve seen in in your body comp changes would be useful.” [HP1]
	Factors affecting receptiveness to body composition feedback	“So they might have [fed back body composition information] I just can’t, I can’t remember to be honest. Yeah, there’s a lot going on. Yeah, it’s quite busy. And sometimes when people try and find you they can’t so they just sort of do it later. And that’s not much, doesn’t happen or whatever and I’m tired. It’s like it’s a lot of learning, probably busy. You know, everyone seems to be busy.” [P3]	Practicality reduced with high patient volume	“..when we …had three or four measurements to try to fit in each week and that was the point where we felt … overwhelmed with how much to try and remember and do.” [HP1]
	Preference for clear visual presentation of body composition results	“….graphs…yeah, it was easy to understand because at least he told you what you were beforehand and what you were after and…[you] got it every month or whatever. That was good. And you could see how you’d gone that month…” [P9]	Preference for clear visual presentation of body composition results	“…the graphs… really helped with translating the data to the patients and explaining it and making it meaningful… and easier to talk about and explain… actually interpreting the trends for them… I found it easier to look at as well and to see what was going on” [HP2]
	Factors affecting food and drink consumption	“I’m not eating as much.. just what I feel like and other than that I don’t realize that I’m not eating at all. I used to eat okay when I first came here, but now I just can’t be bothered. There’s so much other than that [eating and drinking], it doesn’t bother me.” [P2]	Opportunity to learn new skill	“[BIS] was a very new skills for me, so it was good…to be… expanding learning … new skills.” [HP4]

## Discussion

The findings demonstrate that many facets of body composition assessment using BIS are feasible, acceptable and appropriate to patients and clinicians and BIS has the potential to be a useful tool to support patient care and staff clinical decision making. However, low adherence to all components of the pathway suggests that adaptations are needed for improvement, and many such opportunities to improve overall pathway adherence were identified.

Implementing the care pathway into a real-world acute spinal unit presented numerous challenges. Adoption was moderate (62%) due to the high incidence of patients with concomitant acquired brain injury and requiring ventilation. Consequently, the eligibility criteria were modified to include ventilated patients. The 91% consent rate was higher than 80% reported in another study implementing cardiometabolic risk factor monitoring and receipt of health promotion activities in an inpatient rehabilitation SCI centre [27]. Adherence to the dietetic assessment and review components of the pathway was highest at initial assessment (86%) and declined progressively during weeks 2–8 (71%) and >8 weeks post injury (69%). This is likely a reflection of clinical demand exceeding capacity, with allocation of resources to higher acuity/priority patients and competing medical priorities such as patients requiring bedrest for pressure injury management. Similar adherence trends have been reported in another implementation study in head and neck cancer patients [28].

The proportion of patients who had all BIS measurements at all timepoints within pathway timeframes was low (43%) and reflects the complex clinical environment in terms of aligning patient and clinician availability, the need for patients to be supine, able to be weighed and weighed on the same day. The proportion of BIS measurements and energy requirements performed when due was moderate (69%). The main barriers which impeded pathway adherence included patient medical complications, patient and clinician availability, clinician priorities, frequency and number of BIS measurements per week and resources. Time constraints, available resources, insufficient staff and staff turnover have been described as implementation barriers [29]. Although not reflected in the adherence data, staff reported not providing timely body composition feedback to patients due to the multiple steps involved (obtaining weight, entering variables into the computer to calculate body composition, plotting the results, providing patient feedback). This was reflected in patients describing poor communication of their body composition results and the demands, fatigue and cognitive load of rehabilitation affecting their receptiveness to receiving the feedback. However, despite these challenges the results suggest it is feasible to implement body composition assessment using bioimpedance into clinical practice with ongoing support, teamwork, training and simple instructions. Use of a simpler, cheaper, single frequency bioimpedance device may increase uptake at other facilities.

The thematic analysis highlights that patients and clinicians found body composition assessment acceptable and appropriate and helpful in directing the focus of rehabilitation. Care pathways clearly define care expectations for patients and are a means of monitoring progress [29]. This was reflected in patients having an awareness of expected body composition changes, their individual body composition progress and how to optimise their body composition. Patients and clinicians used body composition information for motivation and preferred visual presentation of body composition results (graphs). Patients found body composition results inspiring and clinicians used the detailed body composition information to provide positive reinforcement and to improve patient engagement in physical activity and weight management discussions. The study findings are aligned with Holm et al. [26] who reported that 30% of participants who had DXA measurements, found feedback on DXA motivating for weight management [27]. Dietitians felt that energy requirement calculations were more accurate than weight-based estimates and used the combination of body composition (FFM and % body fat) and energy requirement trends in their clinical decision making and to plan counselling. Similarly, patients described changing food choices and adjusting dietary patterns to minimise weight gain and to optimise muscle such as including additional protein. La Vela et al., [29] found similar nutrition beliefs and eating behaviours in a study of people living with long term SCI [30]. For example, calorie restriction, eating less and changing dietary patterns to manage or control weight; and a larger focus on including protein and fruits and vegetables [30]. Additional benefits of the care pathway included increased frequency of monitoring weight and patients proactively weighing themselves. However, staff acknowledged moral distress when providing body composition feedback to patients with high level tetraplegia due to patients limited ability to engage in physical activity and the role of food as a source of enjoyment. Clinicians felt BIS could be useful to monitor the impact of functional electrical stimulation (FES) cycling on body composition in these patients. Furthermore, body composition information was inaccurate in one undernourished patient. However, overall body composition assessment was considered to be acceptable and appropriate and had a positive impact on patients’ dietary choices.

Implementation and evaluation are a continuous process following introduction of a care pathway [30], hence further adaptations to the care pathway are required. Patients identified a need for more individualised management, detailed and specific advice about portion sizes, calories and more frequent monitoring and feedback. Aligned with the patient feedback, clinicians reported pathway sustainability would be enhanced by limiting body composition measurements to 1–2 patients per week. The adherence data also supports reducing the frequency of BIS assessments, however, further investigation is required to determine if this affects clinical management and outcomes. Other strategies to improve adherence include access to a mobile device to improve efficiency in calculating and graphing body composition results, hence enabling provision of real-time body composition feedback. Prioritising body composition measurements in patients with incomplete injuries and patients with high level tetraplegia interested in FES cycling was also suggested. Hence, there was clear support for ongoing body composition measurement albeit with adaptations to the care pathway.

Study strengths include the pragmatic study design which ensured implementation and evaluation of the care pathway in a genuine clinical setting and the mixed method approach which enabled triangulation of the results. Study limitations include the small sample size, predominantly including individuals with tetraplegia and implementation at a single health service, specialising in acute traumatic SCI. This limits generalisability to persons with non-traumatic SCI and paraplegia. Consequently, confirmation of these findings in a larger patient cohort comprising more individuals with paraplegia and non-traumatic SCI across multiple health care settings is warranted. Other limitations include the BIS measurements were not taken under standardised conditions due to the pragmatic nature of the study [31]. Furthermore, clinicians and patients may not have spoken freely during the interviews and focus group as the study lead was a senior colleague and had a clinical relationship with most patients. The study lead also coded the data therefore bias may have been introduced, however this was minimised during analysis by inclusion of a second coder who was unfamiliar with participants. Furthermore, staffing shortages post COVID-19 may have impacted the delivery and adherence to the care pathway.

This study demonstrates strong support for the feasibility of using BIS to assess body composition and as a useful tool to enable use of SCI-specific energy requirement prediction equations when IC is unavailable in clinical practice. Overall, patients and staff found body composition assessment acceptable and appropriate and beneficial to support patient care, inform dietary choices and staff clinical decision making. Adaptations to the care pathway to improve adherence and further research exploring which patients would most benefit from body composition measures are warranted.

## Supplementary information

Supplementary Table 1

Supplementary Table 2

Supplementary Table 3

Supplementary Table 4

## Data Availability

Deidentified data described in the manuscript will be made available upon reasonable request.
