# Supplementary material for: Implementing body composition assessment into clinical practice in patients with acute spinal cord injury- a pilot feasibility study
Source: Spinal Cord. 2026 Feb 2;64(3):266–78. doi: 10.1038/s41393-026-01169-2 (PMC12975507; doi:10.1038/s41393-026-01169-2)
Supplement: Supplementary file 1 — Supplementary Table 1 [file 41393_2026_1169_MOESM1_ESM.docx]

**Supplementary Table 1.** Summary of the clinical practice guidelines and the recent literature mapped to the AACTT framework

| **Action** | Nutrition assessment, measure energy expenditure using IC or HB 1.1+1.2 & protein 2g/kg & provide nutrition support recommendations (1)  Buchholz et al (2, 3) , SCI-specific energy prediction equation & 1.15 correction factor (4) | Nutrition assessment & implement individualised therapeutic nutrition plan (energy 22.7kcal/kg tetraplegia & 27.9kcal/kg paraplegia & 0.8-1g/kg protein (1)  Buchholz et al (2, 3), SCI-specific energy prediction equation & 1.15 correction factor (4) | Estimate IBW by adjusting metropolitan life insurance tables by 10-15% or 15-20lb tetra & 5-10% or 10-15lb para (1) | Assess body composition using BIA or DXA (1)  BIS and Kocina & Heyward SCI-specific FFM equation (5, 6) | Nutrition assessment for prevention & treatment of overweight & obesity & adjust energy level or implement weight strategies as appropriate (1)  Nutrition education (1)/healthy heart nutrition plan (7) | Assess energy needs/resting metabolism through IC (7, 8)or when unavailable assess RMR/BMR with SCI-specific prediction equation/calculate TDEE using correction factor 1.15 (8) | Assess obesity; measure body composition using 3-or-4 compartment models (7, 8) until validated appropriate equations become available (7)  Classify adult men >22% BF & adults women >35%BF as obese (7) |
| --- | --- | --- | --- | --- | --- | --- | --- |
| **Actor** | Dietitian (1) | Dietitian | Dietitian | Dietitian | Dietitian | Dietitian | Dietitian |
| **Context** | Acute care setting/acute phase (0-4 weeks) (1) | Rehabilitation setting (1) | Not specified | SCI patients who are medically stable (1) | Not specified (1)/after the post-acute period (7) | Not specified | Discharge from rehabilitation & community (7) |
| **Target** | Acute SCI patients | SCI patients | SCI patients | Persons with SCI | Persons with SCI | Persons with SCI | Persons with SCI |
| **Time** | Within 48h post injury & ongoing monitoring (1) | Not specified, should monitor weight, functional capacity, physical activity & adjust energy needs as necessary (1) | Not specified | Not specified  5 weeks | Persons with SCI | Annually (minimally) (8) | At the time of discharge from rehabilitation & at least every 3 years following initial assessment when tests are normal (7) |

IC, indirect calorimetry; HB, Harris-Benedict equation; SCI, spinal cord injury; RMR, resting metabolic rate; BMR, basal metabolic rate; IBW, ideal body weight; BIA, bioimpedance analysis; DXA, dual x-ray absorptiometry; BIS, bioimpedance spectroscopy; FFM, fat free mass; TDEE, total daily energy expenditure; BF, body fat; kcal, kilocalorie; kg, kilogram; lb, pound

1. Academy of Nutrition and Dietetics. Spinal Cord Injury (SCI) Evidence-Based Nutrition Practice Guideline2009 October 2018. Available from: <https://andeal.org/topic.cfm?menu=5292&> pcat=3487&cat=5448

2. Buchholz AC, McGillivray CF, Pencharz PB. Differences in resting metabolic rate between paraplegic and able-bodied subjects are explained by differences in body composition. Am J Clin Nutr. 2003;77(2):371-8.

3. Desneves KJ, Panisset MG, Rafferty J, Rodi H, Ward LC, Nunn A, et al. Comparison of estimated energy requirements using predictive equations with total energy expenditure measured by the doubly labelled water method in acute spinal cord injury. Spinal Cord. 2019;57(7):562-70.

4. Farkas GJ, Gorgey AS, Dolbow DR, Berg AS, Gater DR. Caloric Intake Relative to Total Daily Energy Expenditure Using a Spinal Cord Injury--Specific Correction Factor. Am J Phys Med Rehabil. 2019;98(11):947-52.

5. Kocina P, Heyward V. Validation of a bioimpedance equation for estimating fat-free mass of spinal cord injured adults 319. Med Sci Sports Exerc. 1997;29(5):55.

6. Panisset MG, Desneves K, Ward LC, Rafferty J, Rodi H, Roff G, et al. Bedside quantification of fat-free mass in acute spinal cord injury using bioelectrical impedance analysis: a psychometric study. Spinal Cord. 2018;56(4):355.

7. Nash MS, Sabharwal S, Taylor AJ, Groah SL, Gater Jr DR, Dyson-Hudson TA, et al. Identification and Management of Cardiometabolic Risk after Spinal Cord Injury: Clinical Practice Guideline for Health Care Providers. Lawrence, Kansas: Allen Press Publishing Services Inc.; 2018. p. 379-423.

8. Farkas GJ, Sneij A, McMillan DW, Tiozzo E, Nash MS, Gater Jr DR. Energy expenditure and nutrient intake after spinal cord injury: a comprehensive review and practical recommendations. Br J Nutr. 2022;128(5):863-87.
