# Supplementary material for: Implementing body composition assessment into clinical practice in patients with acute spinal cord injury- a pilot feasibility study
Source: Spinal Cord. 2026 Feb 2;64(3):266–78. doi: 10.1038/s41393-026-01169-2 (PMC12975507; doi:10.1038/s41393-026-01169-2)
Supplement: Supplementary file 2 — Supplementary Table 2 [file 41393_2026_1169_MOESM2_ESM.docx]

**Supplementary Table 2**. Patient semi-structured interview questions and prompts

1. How long you have been living with spinal cord injury.
2. Tell me about your injury, when did it happen and what level is it?
3. Can you tell me how your injury has impacted on your health overall?
4. How has your body composition (amount and proportion of muscle and fat) changed after your spinal cord injury?
5. How do you feel about these changes?
6. What impact (if any) has this had on your function, health and wellbeing?
7. What are the important considerations to you when discussing your nutritional care?
8. How would you describe the nutritional care you received in hospital?
9. Would you have preferred anything to be done differently?
10. Any information you would have liked to be provided that wasn't?
11. Has your experience of SCI changed your approach to nutrition?
12. Could you please tell me your thoughts or how you felt about discussing your body composition (muscle and fat) during dietitian and/or therapy sessions?
13. How was the information presented to you? (Prompts: table, graph, visually, tracking app, other)
14. Did you find it helpful to have information about body composition presented to you? (Prompts: can you explain why?)
15. Did you use information about your body composition (muscle and fat) when making decisions about your diet and/or physical activity with your treatment team? (Prompts: can you explain how?)
16. Do you have any other comments you would like to make regarding your nutritional care or discussions about body composition?
