# Supplementary material for: Implementing body composition assessment into clinical practice in patients with acute spinal cord injury- a pilot feasibility study
Source: Spinal Cord. 2026 Feb 2;64(3):266–78. doi: 10.1038/s41393-026-01169-2 (PMC12975507; doi:10.1038/s41393-026-01169-2)
Supplement: Supplementary file 3 — Supplementary Table 3 [file 41393_2026_1169_MOESM3_ESM.docx]

**Supplementary Table 3**. Staff focus group guide

1. Could you please tell me a little about your role and how long you’ve been working in SCI?
2. Now I would like to ask you about your experience following the care pathway?

*Prompts: impact on patient outcomes & care, perceived burden (effort or time), fit with personal values, understanding of the care pathway and how it works, the benefits of the care pathway, confidence following the care pathway, extent to which the care pathway achieves its purpose (translation of evidenced based care into practice)*

1. Could you please tell me about your experience conducting the body composition measurements?

*Prompts:* *equipment, confidence, ease, time taken, practicalities such as planning the day, electrode availability, patient preparedness*

1. Could you please tell me about your experience in using the body composition information?

*Prompts:* *performing calculations, understanding the results, explaining the results, usefulness of the results, perceived patient understanding*

1. How did you use the body composition results in your clinical decision making?
2. Based on your experience what suggestions/tips do you have for staff providing this information?

*Prompts: amount, trend, interpretation, understanding of information*

1. What do you perceive to be the barriers to using bioimpedance and the energy requirements prediction equation in clinical practice?
2. In your opinion what would be the best ways to overcome these barriers?
3. Do you have any other comments you would like to make regarding your nutritional care or discussions about body composition?
