# Supplementary material for: Implementing body composition assessment into clinical practice in patients with acute spinal cord injury- a pilot feasibility study
Source: Spinal Cord. 2026 Feb 2;64(3):266–78. doi: 10.1038/s41393-026-01169-2 (PMC12975507; doi:10.1038/s41393-026-01169-2)
Supplement: Supplementary file 4 — Supplementary Table 4 [file 41393_2026_1169_MOESM4_ESM.docx]

**Supplementary Table 4** Characteristics of the 12 Patients who participated in Interviews

| **Characteristic** | **Patients** |
| --- | --- |
| Male/Female | 8/4 |
| Age median (IQR) | 47.0 (42.8, 65.3) |
| Days post injury | 13.0 (8.5, 14.5) |
| Injury, n (%) |  |
| High tetraplegia (C1-C4), n (%) |  |
| AIS A/B | 2 |
| AIS C | 4 |
| AIS D | 2 |
| Low tetraplegia (C5-C8), n (%) |  |
| AIS A/B | 2 |
| High paraplegia (T1-T7), n (%) |  |
| AIS C | 1 |
| Low paraplegia (T8-L2), n (%) |  |
| AIS C | 1 |
| Median BMI (IQR) | 24.8 (23.5, 30.1) |

AIS, American Spinal Injury Association (ASIA) Impairment Scale (AIS); BMI, body mass index
